# Ultra-High Field Diffusion MRI Reveals Early Axonal Pathology in Spinal Cord of ALS mice

**DOI:** 10.1186/s40035-018-0122-z

**Published:** 2018-08-08

**Authors:** Rodolfo G. Gatto, Manish Y. Amin, Daniel Deyoung, Matthew Hey, Thomas H. Mareci, Richard L. Magin

**Affiliations:** 1 Department of Anatomy and Cell Biology, University of Illinois at Chicago, 808 S. Wood St. Rm 578 M/C 512, Chicago, IL 60612 USA; 2 Department of Physics, University of Florida, Gainesville, FL USA; 3 Department of Applied Physiology and Kinesiology, University of Florida, Gainesville, FL USA; 4 Department of Biochemistry and Molecular Biology, University of Florida, Gainesville, FL USA; 5 Department of Bioengineering, University of Illinois at Chicago, Chicago, IL USA

**Keywords:** Amyotrophic Lateral Sclerosis, Spinal Cord, Ultra-high Field MRI, Diffusion Tensor Imaging, Yellow Fluorescent Protein, G93A-SOD1 mice, Axonal Degeneration, Tractography, Connectomics

## Abstract

**Background:**

Amyotrophic lateral sclerosis (ALS) is a disease characterized by a progressive degeneration of motor neurons leading to paralysis. Our previous MRI diffusion tensor imaging studies detected early white matter changes in the spinal cords of mice carrying the G93A-SOD1 mutation. Here, we extend those studies using ultra-high field MRI (17.6 T) and fluorescent microscopy to investigate the appearance of early structural and connectivity changes in the spinal cords of ALS mice.

**Methods:**

The spinal cords from presymptomatic and symptomatic mice (80 to 120 days of age) were scanned (ex-vivo) using diffusion-weighted MRI. The fractional anisotropy (FA), axial (AD) and radial (RD) diffusivities were calculated for axial slices from the thoracic, cervical and lumbar regions of the spinal cords. The diffusion parameters were compared with fluorescence microscopy and membrane cellular markers from the same tissue regions.

**Results:**

At early stages of the disease (day 80) in the lumbar region, we found, a 19% decrease in FA, a 9% decrease in AD and a 35% increase in RD. Similar changes were observed in cervical and thoracic spinal cord regions. Differences between control and ALS mice groups at the symptomatic stages (day 120) were larger. Quantitative fluorescence microscopy at 80 days, demonstrated a 22% reduction in axonal area and a 22% increase in axonal density. Tractography and quantitative connectome analyses measured by edge weights showed a 52% decrease in the lumbar regions of the spinal cords of this ALS mice group. A significant increase in ADC (23.3%) in the ALS mice group was related to an increase in aquaporin markers.

**Conclusions:**

These findings suggest that the combination of ultra-high field diffusion MRI with fluorescent ALS mice reporters is a useful approach to detect and characterize presymptomatic white matter micro-ultrastructural changes and axonal connectivity anomalies in ALS.

## Background

Amyotrophic lateral sclerosis (ALS) involves progressive deterioration of upper and lower motor neurons within the brainstem, corticospinal tracts, and anterior horn areas of the spinal cord (SC) [1]. It has been shown that genetic mutations in ALS patients lead to changes in molecular pathways and neuronal degeneration in selective groups of cells subsequently promoting abnormalities in genomic expressions [2] and axonal function [3]. Among the ALS transgenic animal models available, the G93A-SOD1 model is the most widely used to study the neurological deterioration associated with this disease [4].

Unfortunately, other than an increase in the number of available animal models [5–7], little progress has been made toward the early detection of ALS to improve the outcome of therapeutic interventions for these patients. However, a better way to understand the early changes occurring in cellular structures is to include transgenic mouse lines with constitutively expressed fluorescent protein. Thus, a specific neuronal population can be visualized with quantitative fluorescence techniques to assess the morphological aspects of neuronal structures and to understand the role of axonal connectivity in neurological diseases [8–10]. One of the most commonly used fluorescent probes in research is the Thy1-YFP mouse model, expressing yellow fluorescent protein (YFP) in a subset of neurons across different brain structures and axon trajectories in specific layers of the cerebral cortex and spinal cord (SC) [11]. Although the study axonal degeneration with transgenic fluorescent ALS mice has been described in the past [12], such studies were mainly performed at the end stage of the disease with limited focus on changes in the SC white matter (WM) microstructure.

On the other hand, since the average life expectancy of ALS patients from the time of diagnosis ranges from two to five years, any meaningful improvement in the ALS survival rate would depend on the establishment of methods for the detection of early and critical pathogenic events. To this end, magnetic resonance imaging (MRI) provides the best non-invasive way to assess the appearance of neurodegenerative diseases at early presymptomatic stages [13, 14]. In addition, diffusion tensor imaging (DTI) provides substantial information on the organization of neural tissues [15]. As an example, the radial and axial diffusion parameters provide an index of axonal fiber tract integrity and fractional anisotropy gives a measure of brain tissue organization, which is important in the context of ALS. In the search for early ALS biomarkers, recent studies using tractography reconstructions related fiber track changes with a selective degeneration in WM axonal populations [16, 17]. Ultimately, improvements in the sensitivity and specificity of MRI diffusion methods have introduced new imaging biomarkers that could able to identify the changes occurring in the susceptible WM tissues caused by ALS [18].

Although several reports have shown that DTI methods can detect early alterations in WM, and extensive neuropathological evidence points towards early white matter structural alterations in ALS [19–23], the underlying link between the diffusion and the underlying axonal neuropathological process at early stages of the disease has not been fully elucidated. Thus, our study is designed to validate DTI markers obtained by ultra-high field MRI (UHF-MRI) and to characterize early changes in axonal ultrastructure and WM connectivity using an ex vivo tissue and a fluorescent ALS mouse.

## Methods

### Animals

All procedures used to obtain tissues followed an approved protocol from the animal care committee (ACC) at the University of Illinois in Chicago (UIC). C57BJ6 mice, overexpressing the SOD1 transgene with the G93A mutation, were obtained from the Jackson Laboratory (JAX # 004435). The G93A-SOD1 mice have been extensively characterized as an animal model for ALS, developing motor symptoms at approximately 110 days of age and dying around 160 days [24, 25]. Based on these finding, we considered two groups of animals for this work: a presymptomatic group at postnatal day 80 (P80) and a symptomatic group at postnatal day 120 (P120). To evaluate morphologic axonal anomalies in the context of ALS, mice encoding a yellow fluorescent protein (YFP) transgene specifically associated with a neuronal Thy1 promoter was chosen (JAX#003709). Thus, we generated double transgenic mice (YFP, G93A-SOD1) and littermates carrying only the YFP transgene used as a control group. For this study, a total 18 animals (n=5 SCs per P80 and P120 YFP, G93A-SOD1 groups (10 SCs) and n=4 SCs per P80 and 120 YFP control groups (8 SCs), were used for scanning and further histological analysis. Mice had easy access to food and water and were checked daily to assess their level of well-being and health. In any situation of animal distress or pain, animals were euthanized in carbon dioxide using standard protocols.

### Animal preparation for MRI imaging

Animals were rendered unconscious with CO_2_ inhalation, then transcardiac perfused with a PBS and 4% paraformaldehyde (PFA) solution. A laminectomy was performed and the spinal cords were extracted intact then immersed in PFA (> 48 hours). Prior to imaging, the cords were soaked overnight in PBS to removed free fixative, then placed in individual 5 mm diameter NMR tubes (New Era #NEML5-7, 300-400 MHz) filled with fluorocarbon oil (Fluorinert®, 3M, Maplewood, MN). Each set of 9, 5-mm tubes (each containing a spinal cord) was positioned in a 20-mm diameter NMR tube (New Era # NE-L25-7) using a custom-made plastic tube holder. Images were acquired with a 17.6T vertical-bore Avance II scanner using a 20-mm RF coil, Micro-2.5 gradients, and Paravision 6.0 software (Bruker, Karlsruhe, Germany).

### Diffusion weighted imaging and data processing

For each set of 9 spinal cords (2 imaging sessions) a total of 60 MRI slices were acquired in blocks of 20 slices, centered at the cervical, thoracic, and lumbar levels each, and oriented along the rostral-caudal axis of the spinal cord. Diffusion weighted images were acquired using a spin echo sequence with TR= 4000 ms and TE =28 ms, interleaved 0.15 mm thick slices, field of view = 20 x 20 x 3 mm^3^ in each block of slices, in-plane acquisition matrix = 133 x 133, for an isotropic image resolution of 150 μm. For connectomics calculations ([26]), two images were acquired with b = 0 s/mm^2^ and diffusion weighting was applied with b = 700 s/mm^2^ in 12 directions, and b = 2500 s/mm^2^ in 64 directions [27], with 3.5 ms gradient pulses and 17.5 ms separation. This acquisition was averaged twice for a total acquisition time of 19 hrs. per set.

**Fig. 1:**
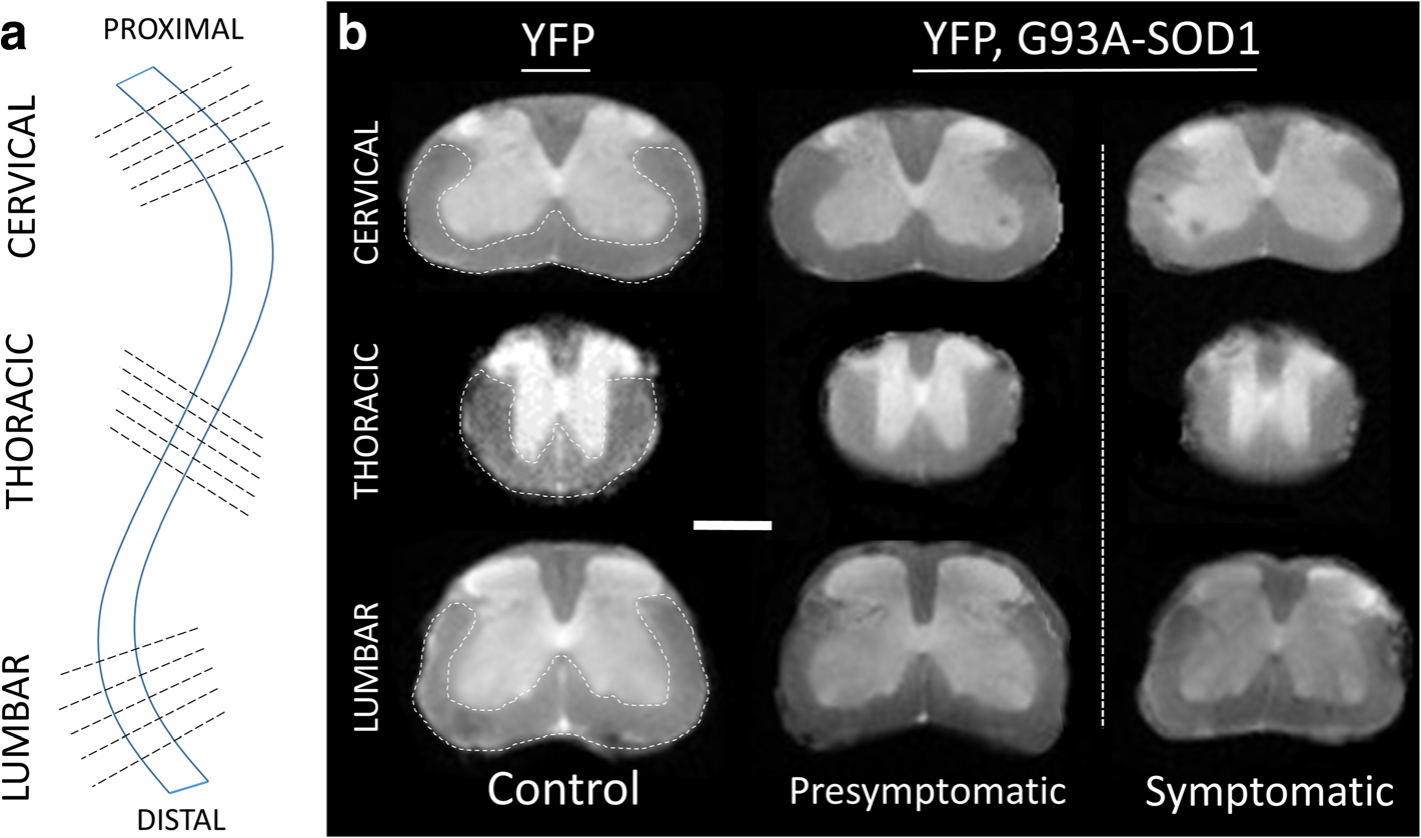
Ex vivo analysis of presymptomatic spinal cord of ALS mice by Ultra-High Field MRI diffusion. **a** Scheme representing MRI cross- sections from different spinal cord segments (cervical, thoracic, and lumbar) used for analysis. **b** T2w representative MR images from individual spinal cords scans showing the white matter (WM) ROIs in the WM anterolateral funiculus (white dotted line) from each spinal cord segment (YFP vs. G93A-SOD1 mice) Scale bar = 1 mm. Abbreviations: WM: white matter. YFP: yellow fluorescent protein

Diffusion data processing was performed using FSL [28] to calculate fractional anisotropy (FA), axial diffusivity (AD), and radial diffusivity (RD) [29]. The average apparent diffusion coefficient (ADC) over the 64 directions was calculated using b = 0 and b = 2500 s/mm^2^. In addition, the calculation of the water displacement probability density function [30] was used to estimate white matter tracts. White matter regions-of-interest (ROIs), defined within slices at the top and bottom of each of spinal cord segments (cervical, thoracic, and lumbar), were manually outlined following the anterolateral distribution of the white matter on each slice (Fig. 1) using ImageJ software (NIH, Bethesda).

### White matter connectivity

To determine white matter structural connectivity in the three regions of the spinal cords, deterministic tractography was performed using in-house software using 125 seeds per voxel, an angular threshold of 50^o^, and a step size of 75 μm. Fiber tracks were visualized with Trackvis (Version 0.6.1, Massachusetts General Hospital, Boston, MA). Using a network approach to define white matter integrity along the spinal cord, the top and bottom slices in each segment were used to define network nodes. White matter tracts were used to define network edges and the connectivity quantified using a dimensionless, scale-invariant edge weight (EW) [26].

### Histology and immuno-fluorescence analysis

**Fig. 2:**
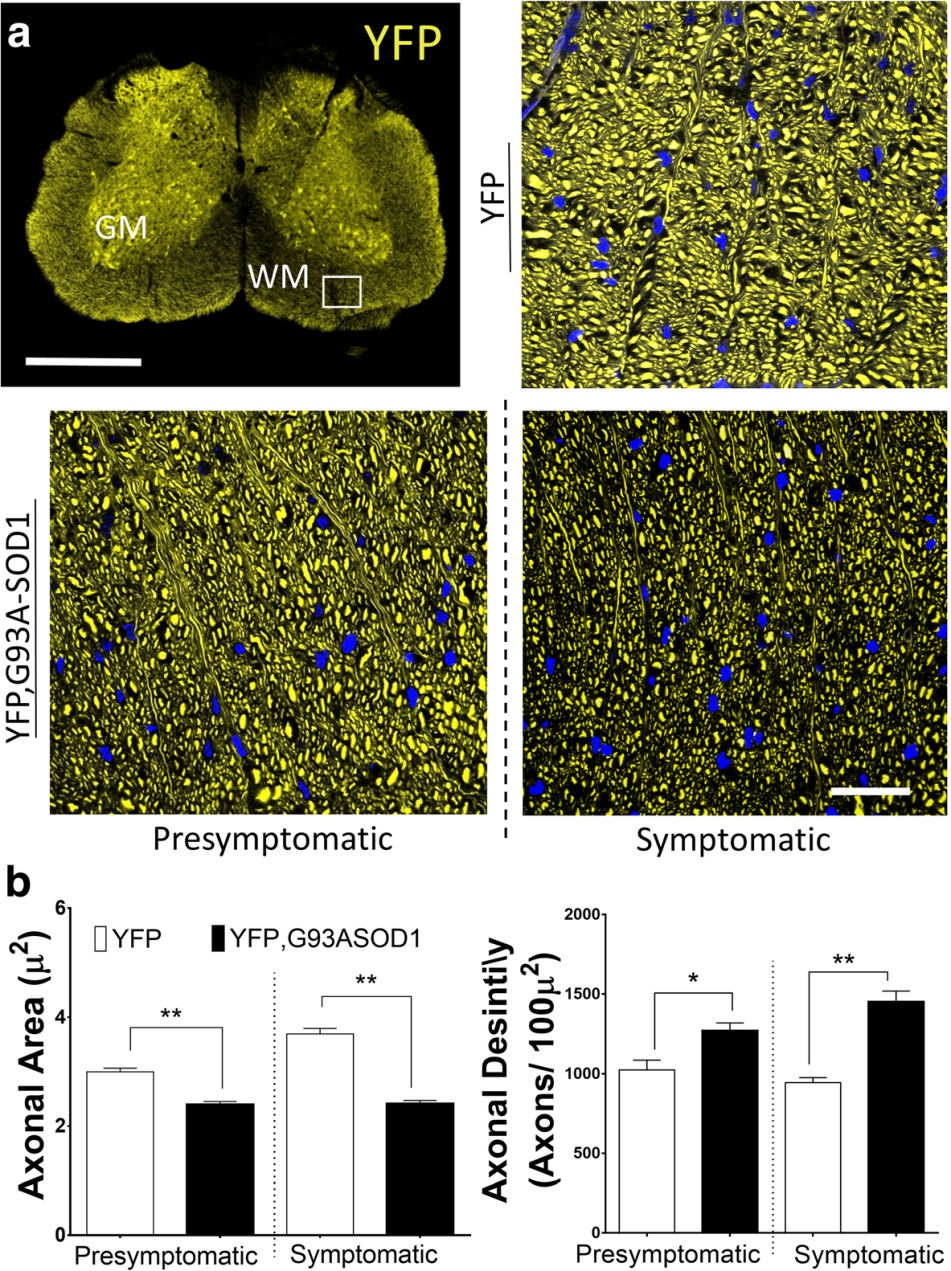
Early axonal structural changes can be observed in spinal cord of ALS mice. **a** Spinal cord lumbar sections from an YFP, G93A-SOD1 mouse. Regions of Interest (ROI) were obtained from the anterior portion of the spinal cord (SC) white matter (WM) (white square area). Detailed changes in axonal diameter can be seen in the YFP, G93A-SOD1 mice. Note that each WM area in the histology pictures has an approximate voxel size (100 × 100 μm^2^^)^. Scale bar =1 mm. **b** Measurements from WM ROIs showed a significant (**p < 0.01) reduction in in axonal areas and an increase in the number of YFP-positive axons (axonal density contained within 100 μm^2^) in the presymptomatic (P80) YFP, G93A-SOD1 mice. (* = *p* < 0.05); (** = *p* < 0.01) (*n* = 4/5 per group). Nuclear counterstaining with DAPI (blue). Scale bar =10 μm. Abbreviations: WM, white matter; GM, grey matter; YFP, yellow fluorescent protein

Although it is feasible to histologically examine all segments of the spinal cord (cervical and thoracic and lumbar), results from this and previous MRI diffusion ex-vivo studies [31] have shown predominant alterations in MRI diffusion at the lumbar segments of ALS mice. Thus, this study is focused in the histological analysis of white matter anomalies in the lumbar regions, specifically between the third and fifth lumbar segments (Fig. 2).

After MRI scanning, oil media was removed and spines were placed in progressive solutions of sucrose [5-30 %] for an additional 24 hrs for cryo-protection. After embedding in optical cutting temperature (OCT) polymer compound (Tissue Tek, Sakura, Finetek, cat #4583), 50 μm thick spinal cord sections were obtained using a microtome (Leica cryostat CM 1850 Cryostat, Buffalo Grove, IL). Based in our previous finding to detect early SC white matter (WM) changes [32], only coronal section of lumbar spinal cord were used for histological analysis. Spinal cord sections were mounted on slides (Fisher-brand Superforst, cat# 12-550-15) and dried for 15 minutes. Then, the OCT was removed by washing three times with Tris base buffer (TBS). Sections were permeabilized with Triton-X100 0.25 % for 10 minutes and blocked with 5 % goat serum for an hour in TBS. Spinal cord sections (50μm thickness) were mounted on slides. Slides were dried and mounted in Vecta-Shield mounting media (Vector Laboratories, Burlingame, CA). Images were acquired by confocal microscopy (Leica LMS-710 confocal microscope, Germany). Each coronal section in the lumbar spinal cord was selected using similar anatomical reference following a spinal cords stereotaxic coordinates (The Spinal Cord: A Christopher and Dana Reeve Foundation Text and Atlas, 1st Ed, Watson & Paxinos, 2008). Confocal microscopy images and z stack images for three-dimensional reconstruction were obtained by background subtraction using negative controls samples without primary antibody and collected by two independent channels: 534 nm channel for the YFP yellow signal and 647 nm channel to detect fluorescent emission from antibodies from other markers. To evaluate the role of water permeability in ALS, we used anti aquaporin-4 staining (AQP4) (StressMarq Bioscence Inc., Victoria, BC) (Cat #SPC-505D, 1:400). Quantitative measures were obtained by counting the mean pixel value per equal picture area using auto-threshold methods and the pixel aggregates of each figure compiled and tabulated for analysis. Briefly, the procedure divides the image into objects and background by taking an initial threshold. Averages of the number of pixels at, below, or above the threshold were computed and subsequent averages of these two values were used.

### Statistical analysis

Quantitative data were tabulated and analyzed using GraphPad Prism 6 software (La Jolla, CA). Based on the results from pilot experiments, the group size of animals per experimental group were established using power analysis and sample size calculations. For quantitative analysis of YFP, and AQP4 fluorescence levels and MRI diffusion values (ADC, FA, AD and RD), one-way ANOVA and Tukey’s post hoc tests were used to determine statistical differences among experimental animal groups (P80 and P120). A value of p < 0.05 was used to demonstrate statistical significance. Results were replicated by application of non-parametric statistical tools (Mann–Whitney test). Error bars in all the figures represent standard error of the mean (SEM).

## Results

### MRI diffusion demonstrate presymptomatic spinal cord changes of YFP, G93A-SOD1 mice

Previous clinical and animal diffusion imaging studies ALS SCs have shown that regional changes in parameters were more affected at the distal than proximal regions [14, 33]. Hence, we centered our MRI diffusion studies on the anterolateral white matter funiculi of three SC regions (cervical, thoracic and lumbar segments) (Fig. 1a). Manual segmentation of WM ROIs across diffusion maps from five SC slices were considered in this analysis, (Fig. 1b). Two time-points were considered; a presymptomatic stage (P80) and a symptomatic stage (P120). At presymptomatic stages (P80), we observed a significant reduction of FA in the cervical region (*p* < 0.05) in the YFP, G93A-SOD1 mice (YFP mice =0.60 +/- 0.01 versus YFP, G93A-SOD1 mice = 0.57 +/- 0.01) (- 6.6%) as well as in the thoracic regions (*p* < 0.01) (YFP mice = 0.66 +/- 0.01 versus YFP, G93A-SOD1 mice = 0.58 +/- 0.01) (- 12.6%) and lumbar region (*p* < 0.001) (YFP mice = 0.66 +/- 0.01 versus YFP, G93A-SOD1 mice = 0.53 +/- 0.03) (- 19.7%). A significant decrease in axial diffusion (AD) in the lumbar segment was seen at P80 (YFP mice = 6.7 +/- 0.1x10^-4^ mm^2^/s versus YFP, G93A-SOD1 mice = 6.3 +/- 0.05x10^-4^ mm^2^/s) (*p* < 0.01) (-8.7 %). These changes were decreased further on each spinal cord segment during the symptomatic stage. Presymptomatic results in radial diffusion (RD) demonstrate a significant (*p* < 0.01) increase not only in the lumbar levels (YFP mice = 2.0 +/- 0.1x10^-4^ mm^2^/s versus YFP,G93A-SOD1 mice = 2.7 +/- 0.5x10^-4^ mm^2^/s) (*p* < 0001) (+35 %) in the dorsal region (YFP mice = 2.5 +/- 0.1x10^-4^ mm^2^/s versus YFP,G93A-SOD1 mice = 3.1 +/- 0.1x10^-4^ mm^2^/s) (*p* < 0001) (+24.8%) and cervical levels (YFP mice = 2.2 +/- 0.2x10^-4^ mm^2^ /s versus YFP, G93A-SOD1 mice = 2.52 +/- 0.9x10^-4^ mm^2^/s) (p<001) (+16.1 %). Overall, these DTI findings demonstrated that lumbar changes in diffusion across axonal structures in this ALS mice can be detected before symptoms manifest (Fig. 4a).

### Presymptomatic changes in white matter diffusion are linked to axonal structural anomalies

Alterations in axonal features from spinal cords (SC) in G93A-SOD1 mice associated to a fluorescent reporter has been previously reported [12]. However, the results from these studies only carried descriptive findings and no specific quantitative analysis has been done. To determine the structural changes underlying the WM alterations in diffusion observed in previous MRI scans, we focused our histological analysis on the anterolateral region at the lumbar SC section from YFP and YFP, G93A-SOD1 mice (Fig. 2a). To make results equivalent to the voxel size performed during the MRI sessions (voxel size = 100 x100 μm^2^) we performed structural analysis using confocal fluorescence images with similar size. Specifically, for morphological evaluations we manually registered WM ROIs for each animal and group following similar topography and guided by stereotaxic coordinates [34]. Measurements from each groups showed a significant (*p* < 0.01) reduction (-22.1 %) in axonal areas in the YFP, G93A-SOD1 mice at P80 (YFP mice = 2.9 +/- 0.06 μm^2^ versus. YFP, G93A-SOD1 mice= 2.3 +/-0.05 μm^2^) and -41.9% P120 (YFP mice = 3.69 +/-0.09 μm^2^ versus YFP, G93A-SOD1 mice = 2.4 +/-0.05 μm^2^) (*n* > 2000 axons per group)**.** Nonetheless, we also observed a significant (*p* < 0.05) increase in the number YFP-positive axons delimited within each ROI (Axonal Density) in the YFP, G93ASOD1 mice group (+21.6 %) at P80 (YFP mice 1024 +/- 61 axons/100μm^2^ vs YFP, G93A-SOD1 mice 1272 +/- 48 axons/100μm^2^) and more significant (*p* < 0.01) increase (+42.4 %) at P120 (Fig. 2b). All together, these results point towards a significant remodeling of the SC axonal structures at early stage of the disease.

### Presymptomatic structural changes in axonal fibers are associated with alterations in axonal connectivity

**Fig. 3:**
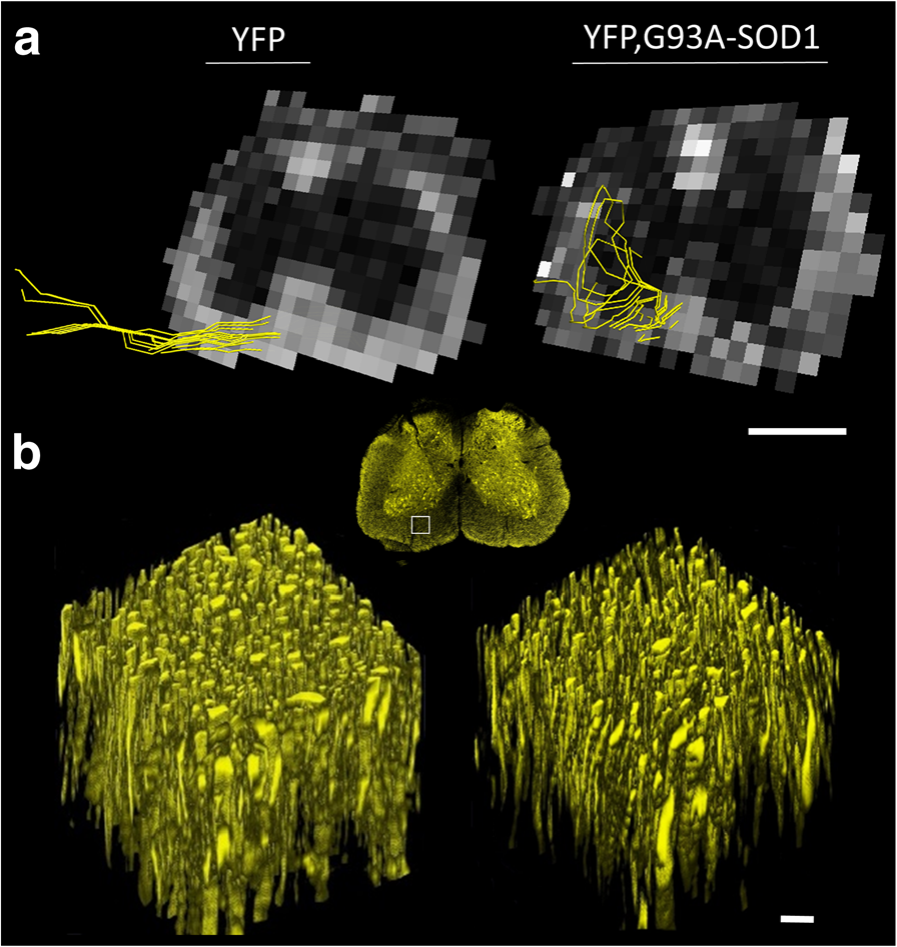
Fiber organization tractography anomalies and ultrastructural histological changes in axons from ALS mice. **a** Representative WM tractography fiber reconstructions from lumbar spinal cord sections showing early changes in microstructural organization. Scale bar =1 mm. **b** Three-dimensional confocal z-stack reconstructions from spinal cord WM (white square) showing early axonal structural anomalies in the YFP, G93A-SOD1 (ALS) mice. Scale bar =10 μm. Abbreviations: WM, white matter; YFP, yellow fluorescent protein

**Fig. 4:**
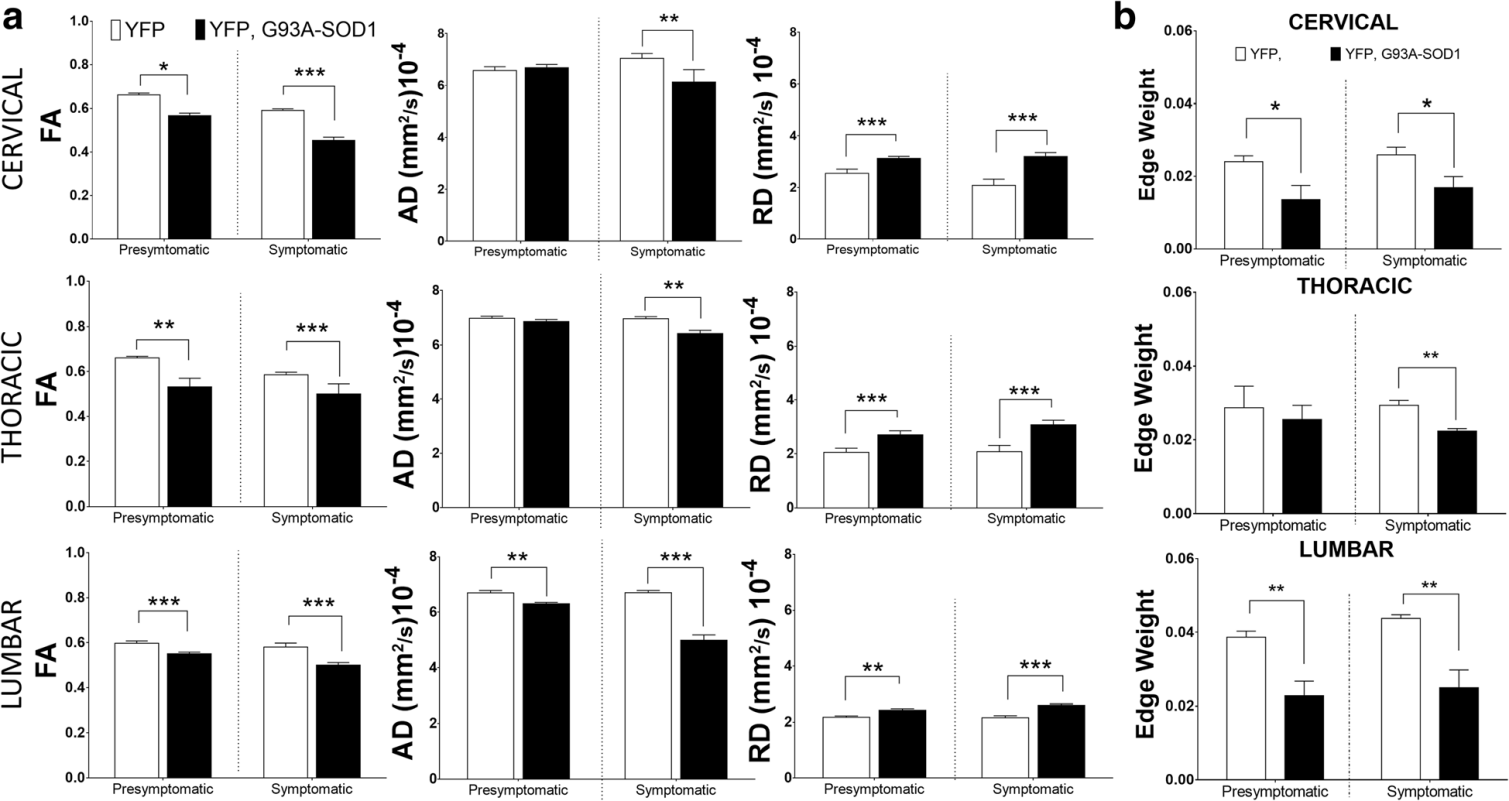
Presymptomatic white matter fiber from the YFP, G93A-SOD1 mice are associated to quantitative changes in axonal connectivity. **a** Quantitative analysis of Fractional Anisotropy (FA), Axial Diffusion (AD) and Radial Diffusion (RD) from spinal cord (SC) white matter (WM) showing a significant decrease in FA and AD and an increase in RD in the YFP, G93A-SOD1 (ALS) mice compared with controls (*p < 0.05) (**p < 0.01), (***p < 0.001) (*n* = 4/5). **b** Statistical analysis of Edge Weight analysis from ROIs showing statistical differences in presymptomatic (P80) WM connectivity in the YFP, G93A-SOD1 mice compared with controls. (* = *p* < 0.05), (** = *p* < 0.01). Abbreviations: YFP, yellow fluorescent protein; P80, postnatal day 80 (presymptomatic)

Using tractography reconstructions and histological confocal reconstructions we have demonstrated a critical impact of ALS towards axonal connectivity. Specifically, tractography methods showed early anomalies in fiber organization in the YFP, G93A-SOD1 mice and histological reconstructions evaluated the specific structural anomalies across individual axons (Fig. 3a and b). Among new techniques has been developed for quantitative connectome analysis [35], edges weights (EW) analysis has been introducing the concept of the adjustment and quantitation of connection strengths [36] as calculated from our data set (Fig. 4b). Results from this analysis showed an early significant decrease of this parameters (*p* < 0.01) at P80, at the cervical levels (YFP mice = 0.024 +/- 0.002 versus YFP, G93A-SOD1 mice = 0.0136 +/- 0.004) (*p* < 0.05) (-43.5 %), as well as in the SC lumbar regions (YFP mice = 0.039 +/- 0.0001 versus YFP, G93A-SOD1 mice = 0.023 +/- 0.0004) (p<0.01) (-40.9 %), (Fig. 4c).

### Early ADC anomalies in the spinal cord of the ALS mice are coupled with changes in water transport protein membranes

**Fig. 5:**
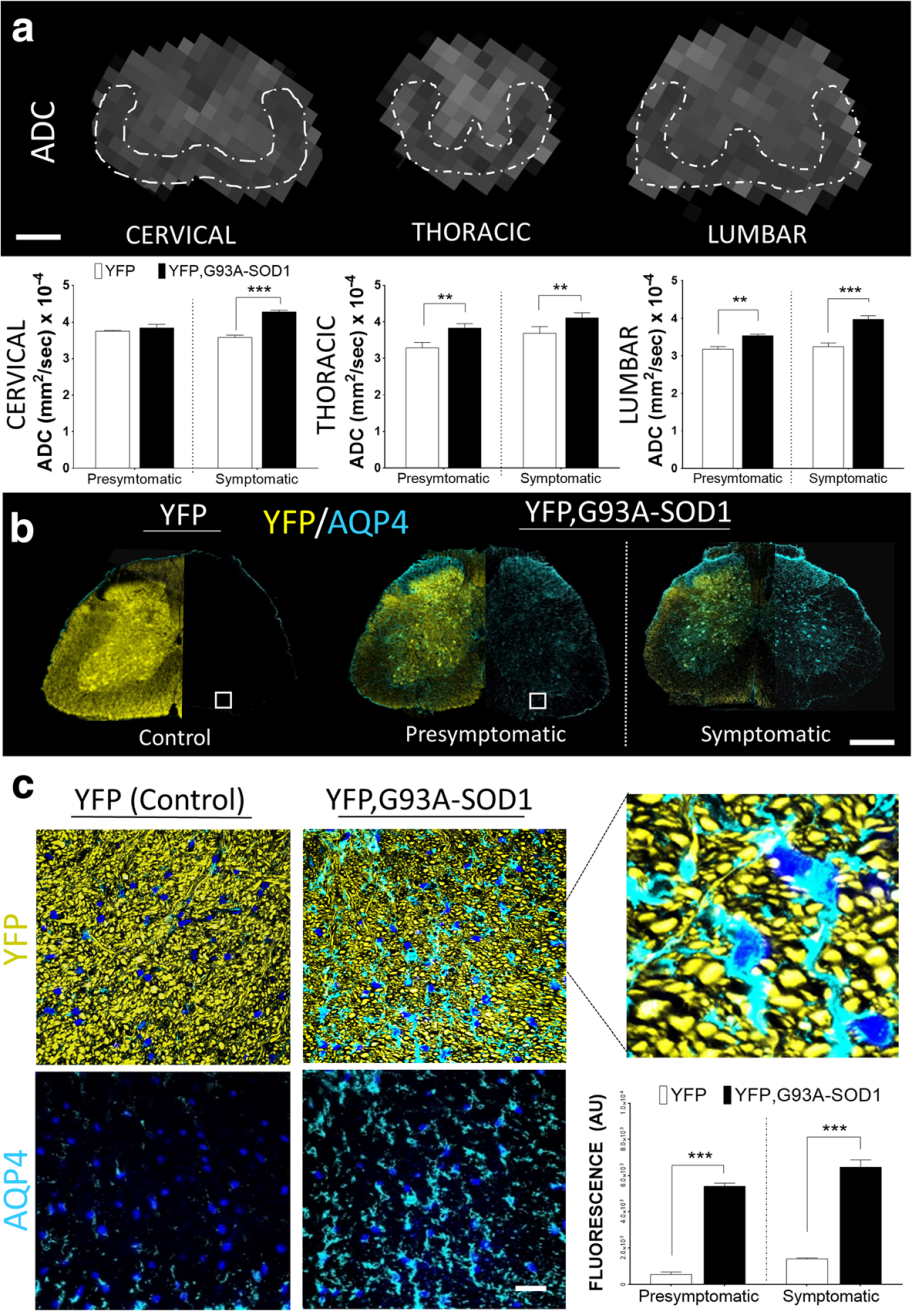
Changes MRI diffusion are coupled with aquaporin expression anomalies in ALS white matter. **a** Quantitative measurements from apparent diffusion coefficient (ADC) spinal cord maps showing an increase in lower SC segments (dorsal and lumbar levels) in the ALS mice. **b** Progressive changes in aquaporin 4 (AQP4) expression can be seen in the presymptomatic and symptomatic YFP, G93A-SOD1 mice. Scale bar = 1 mm. **c** Further analysis of SC white matter (WM) shows a significant increase in AQP4 expression in the presymptomatic ALS mice compared to control, mostly located in the extra- axonal compartment. (*** = *p* < 0.001). Nuclear counterstaining with DAPI (blue). Scale bar = 10 μm. Abbreviations: YFP, yellow fluorescent protein; P80, postnatal day 80 (Presymptomatic); P120, postnatal day 120 (Symptomatic)

The role of ADC in SC ALS tissue has been described before but it changes in the early stages of the disease remain unknown [16, 37]. Measurements from our experimental model have shown early changes of this parameter particularly at the thoracic (YFP mice = 3.2 +/- 0.1x10^-4^ mm^2^ /s versus YFP, G93A-SOD1 mice = 3.8 +/- 0.1x10^-4^ mm^2^/s) (p<001), (+16.4 %) and lumbar level (YFP mice = 3.2 +/- 0.1x10^-4^ mm^2^ /s versus YFP, G93A-SOD1 mice = 3.53 +/- 0.9x10^-4^ mm^2^/s) (p<001) (+23.3 %) (Fig. 5a). The complex nature of diffusional water exchange in diverse neuropathological conditions is associated to changes in membrane permeability [38, 39]. One of the factors regulating this water diffusion process is determined by the transmembrane proteins (aquaporin channels) [40]. Aquaporin 4 (AQP4) has shown a high prevalence in the central nervous system (CNS). Quantitative IHC analysis revealed a significant (*p* < 0.001) increase of AQP4 in the ALS mice SC at the presymptomatic stage (P80) (YFP mice = 541.6 +/- 131.5 a.u. vs YFP, G93A-SOD1 mice = 5403 +/-176 a.u.) (1-2 fold increase) predominantly in the extra-axonal compartment (Fig. 5b and c).

## Discussion

ALS is characterized by a selective loss of motor neurons in the brain and spinal cord [41]. Although the underlying mechanism of the disease is unknown, pathological observations and experimental data establish that alterations in synaptic and neuronal function occur well before neuronal death [41], supporting the principal role of axonal degeneration in the neuropathological process [3]. The earlier compromise of motor axons, and alterations in water diffusion in the spinal cords from an animal model of ALS have been increasingly gaining attention [42–44]. An unproven hypothesis of early axonal structural changes has been proposed to explain the preponderance of early phenotype and symptoms in ALS and other neurodegenerative diseases [10, 45]. Furthermore, our previous MRI studies demonstrated that early axonal injury in the G93A-SOD1 mice model occurred at the lower level of the spinal cord and that such microstructural changes could be monitored using DTI [31]. Moreover, *in vivo* UHF-MRI diffusion studies have a critical advantage for longitudinal studies acquired with high spatial resolution [46]. From the medical perspective, using DTI to gain an understanding of the ultrastructural changes occurring during the early stage of ALS may improve the detection and treatment of this disease at earlier stages [10, 47].

Previous work G93ASOD1 mice were not able to demonstrate presymptomatic changes in DTI diffusion parameters using 7T and 9.4T MRI instruments in combination with conventional histology [18, 48–52] (Table 1). To the best of our knowledge, this study is the first to use UHF-MRI at 17.6T to interrogate the WM microstructure of the spinal cord from an ALS mouse model. The combination of higher signal to noise ratios (SNR) available at higher magnetic fields [53, 54], and the histological detail provided by an ALS transgenic mice expressing a neuronal specific endogenous fluorescent protein (YFP), enhanced the detection of early changes in DTI parameters and WM microstructure (Figs. 1b and 4a). Thus, we have identified presymptomatic changes in MRI diffusion are based on alterations in of morphological axonal features, such as a reduction in axonal areas and increase in axonal density (Fig. 2).

**Table 1:** Previous Spinal Cord Studies in ALS Using Diffusion Tensor Imaging (DTI)

Research group		Experimental Setup					Diffusion Coefficients			
Author	Year	Animal Models & Subjects	Cohort	MRI instrument	ROI	SC Level	FA	AD	RD	MD
Gatto et al. [28]	2018	B6JL G93A-SOD1 P40 (PS)	G93A-SOD1 (n = 5) WT (n = 5) (Ex-Vivo)	9.4 T Bruker Agilent	WM(ALF) GM	C,D,L	Decrease(WM) Increase(GM)	Decrease(WM) Increase (GM)	Increase(WM) Decrease(GM)	Increased(WM) Decrease(GM)
Fukuri et al. [81]	2017	Clinical	ALS patients (n = 38) Patient controls (n = 8)	3 T Siemens MAGNETROM	WM	C5	Decrease	Not Evaluated	Not Evaluated	Unchanged
Mancuzzo et al. [15]	2017	B6JL G93A-SOD1 P50,P56,P70(PS) & P84,P105,P128 (S)	G93A-SOD1 (n = 7) WT (n = 7) (In-Vivo)	7 T Bruker BioSpec	D,DR,DL,V,VR,VL	L	Decrease	Unchanged (PS) Decrease (S)	Unchanged	Decrease
Rasoanandrianina et al. [82]	2017	Clinical	ALS patients (n = 10), Patient controls (n = 20)	3 T Siemens MAGNETROM	WM (CST, PST) GM	C2-C5	Decrease WM	Decrease(WM) Increase (GM)	Increase(WM) Increase(GM)	Not Evaluated
Budrewicz et al. [83]	2016	Clinical	ALS patients (n = 15), Patient controls (n = 10)	1.5 T GE Medical systems	ACST,PCST, LCST, GM	C1-C5	Decrease	Not Evaluated	Not Evaluated	Not Evaluated
Figini et al. [17]	2016	B6JL - G93A-SOD1 P70 (PS) & P119 (S)]	G93A-SOD1 (n = 7) vs WT-SOD1 (n = 7) (In-Vivo)	7 T Bruker BioSpec	VMT, dT, VLT, DLT, GM	L	Decrease	Unchanged (PS) Decrease (S)	Increase	Increase
Caron et al. [46]	2015	129Sv G93A-SOD1 P105,P133,P154 (S) & C57 SOD1-G93A P44 (PS); P105,P133,P154 (S)	C57 G93A-SOD1 (n = 6) 129Sv G93A-SOD1(n = 5) WT (n = 6,5) (In-Vivo) & C57 G93A-SOD1 (n = 16) 129Sv G93A-SOD1(n = 16) WT(n = 16) (Ex-Vivo)	7 T Bruker BioSpec	VMT,VLT,DLT, dT	L	Decrease	Unchanged (PS) Decrease (S)	Increase	Not Evaluated
Iglesias et al. [84]	2015	Clinical	ALS patients (n = 20) Patient controls (n = 19)	3 T Siemens	PMT, CST	C5- D1	Decrease	Unchanged	Increase	Increase
Wang et al. [10]	2014	Clinical	ALS patients (n = 24), Patient controls (n = 16)	1.5 T GE healthcare	ST, LCST	C1-C2	Decrease	Not Evaluated	Not Evaluated	Not Evaluated
Romano et al. [85]	2014	Clinical	ALS patients (n = 14) Patient controls (n = 14)	1.5 T Siemens	CST	C,D,L	Decrease	Unchanged	Increase	Increase
El MendilI et al. [86]	2014	Clinical	ALS patients (n = 14) Patient controls (n = 15)	3 T Siemens Trio	CST	C2 to T6	Decrease	Unchanged	Increase	Increase
Cohen- Adad et al. [87]	2013	Clinical	ALS patients (n = 29) Patient controls (n = 21)	3 T Siemens Trio	PSCT, LSCT	C2 to T2	Decrease	Decrease/Unchanged	Increase	Not Evaluated
Kim et al. [18]	2011	B6JL G93A-SOD1 P84 (S)	G93A-SOD1 (n = 5) WT (n = 5) (In-Vivo)	4.7 T Oxford Instruments	VLT, dT	C & L	Decrease	Decrease	Increase	Not Evaluated
Underwood et al. [12]	2011	C57 G93A-SOD1 P145 (S)	G93A-SOD1 (n = 6) G93A-SOD1 (n = 6) (In-Vivo)	16.4 T Bruker	DF,VF,VLF,DLF	D12-L1	Decrease	Decrease	Increase	Not Evaluated
Nair et al. [11]	2010	Clinical	ALS patients (n = 14) Patient controls (n = 15)	3 T Siemens Trio	CST	C1-C6	Decrease	Unchanged	Increase	Increase
Agosta et al. [30]	2009	Clinical	ALS patients (n = 17) Patient controls (n = 20)	1.5 T Siemens Avanto	CST	C2-C3	Decrease	Not Evaluated	Not Evaluated	Increase
Valsalina et al. [88]	2007	Clinical	ALS patients (n = 28) Patient controls (n = 20)	1.5 T Siemens Avanto	CST	C2-C3	Decrease	Not Evaluated	Not Evaluated	Decrease
Rossi et al. [89]	2007	Clinical	ALS patients (n = 1) Patient controls (n = 8)	1.5 T and 3 T Scanner	CST	C	Decrease	Not Evaluated	Not Evaluated	Decrease

Summary of previous studies using DTI to investigate microstructural changes in ALS spinal cord

Abbreviations*: FA* Fractional Anisotropy *SC* Spinal Cord, *C* Cervical segment, *D* Dorsal segment, *L* Lumbar segment, *RD* Radial Diffusion, *AD* Axial Diffusion, *MD* Mean Diffusion, *ROI* Region of interest, *CST* Corticospinal Tracts, *LCST* Lateral Corticospinal Tracts, *PCST* Posterior Corticospinal Tract, *ACST* Anterior Corticospinal Tract, *CMG* Central Grey matter, *ST* Spinothalamic Tract, *PMT* Posterior Medial Tract, *CMG* Central Grey Matter, *ST* Spinothalamic Tracts, *PMT* Posterior Medial Tract, *VMT* Ventromedial Tract, *PS* Presymptomatic, *S* Symptomatic, *dT* Dorsal Tract, *VLT* Ventrolateral Tract, *DLT* Dorsolateral Tract, *DF* Dorsal Funiculi, *VF* Ventral Funiculi, *VLF* Ventrolateral Funiculi, *DLF* Dorsolateral Funiculi.[Source: PubMed. Key words: ALS / DTI /Spinal Cord /Diffusion Tensor imaging]

Besides the increased number of mechanisms responsible for the alteration of axonal function in ALS [3, 55, 56], the microstructural alterations causing the changes the diffusion signal in ALS are still not well-known. Changes in axonal are widely used in diverse scientific and clinical scenarios [57–59]. Nonetheless, such tractography features are an under representation of the real neuropathological changes occurring inside a single voxel (Fig. 3). Thus, such non-quantitative parameters have to be perfected and validated before can be used to make further decisions in clinical scenarios [60–63].

Although the micro-anatomical anomalies displayed in this work could explain the early changes in diffusion in ALS, the analysis of axonal connectivity could unveil a further insight in the real axonal disconnection problem observed in many neurodegenerative diseases [57, 59]. Obtained by diffusion path probabilities, such techniques can estimate the connection strengths across different WM regions (nodes) enabling the reconstruction of connectomes, consistent with CNS networks properties, mapped by previous imaging modalities and post-mortem brain studies [64]. Using quantitative techniques measuring the diffusion properties along edge voxels, such as edge weight (EW) analysis [26, 36], we evaluated the early axonal disconnection in this animal model (Fig. 4b). Specifically, we addressed how structural information across spinal cord axons was linked and early impacted by ALS. These results have pointed to an early disconnection predominantly in cervical and lumbar regions, as observed in two thirds of ALS patients with upper and lower limbs symptoms (spinal form) [65].

In addition to the microstructural changes observed during early stages, another point of attention are the events related to alteration in cellular and membrane water exchange and their relationship with changes in MRI diffusion signals [66–68]. One of the mechanisms to maintain the osmotic balance across cellular membranes in the central nervous system is regulated by an intricate mechanism of water permeably controlled by membrane channels called Aquaporin (AQP) [69–73]. Although many structural alterations can be the cause of changes in water diffusion across cellular membranes, recent work has shown a significant link between AQPs and the MRI apparent diffusion coefficient (ADC) [67]. Although such parameter has been found highly impaired in ALS models [74], changes in ADC has not be fully characterized in early stages of ALS. In that regard, our studies have demonstrated that changes in AQP4 and ADC are present at earlier stages of the disease (Fig. 5a and b). Moreover, based in our histological finding such increase in AQP4 channel expression could reveal additional anomalies across different cellular types located in the non- axonal compartment (Fig. 5c). Nevertheless, the specific role of AQP channels and their contribution to the water exchange and MRI diffusion in the early stages of ALS remains to be determined [75, 76].

**Fig. 6:**
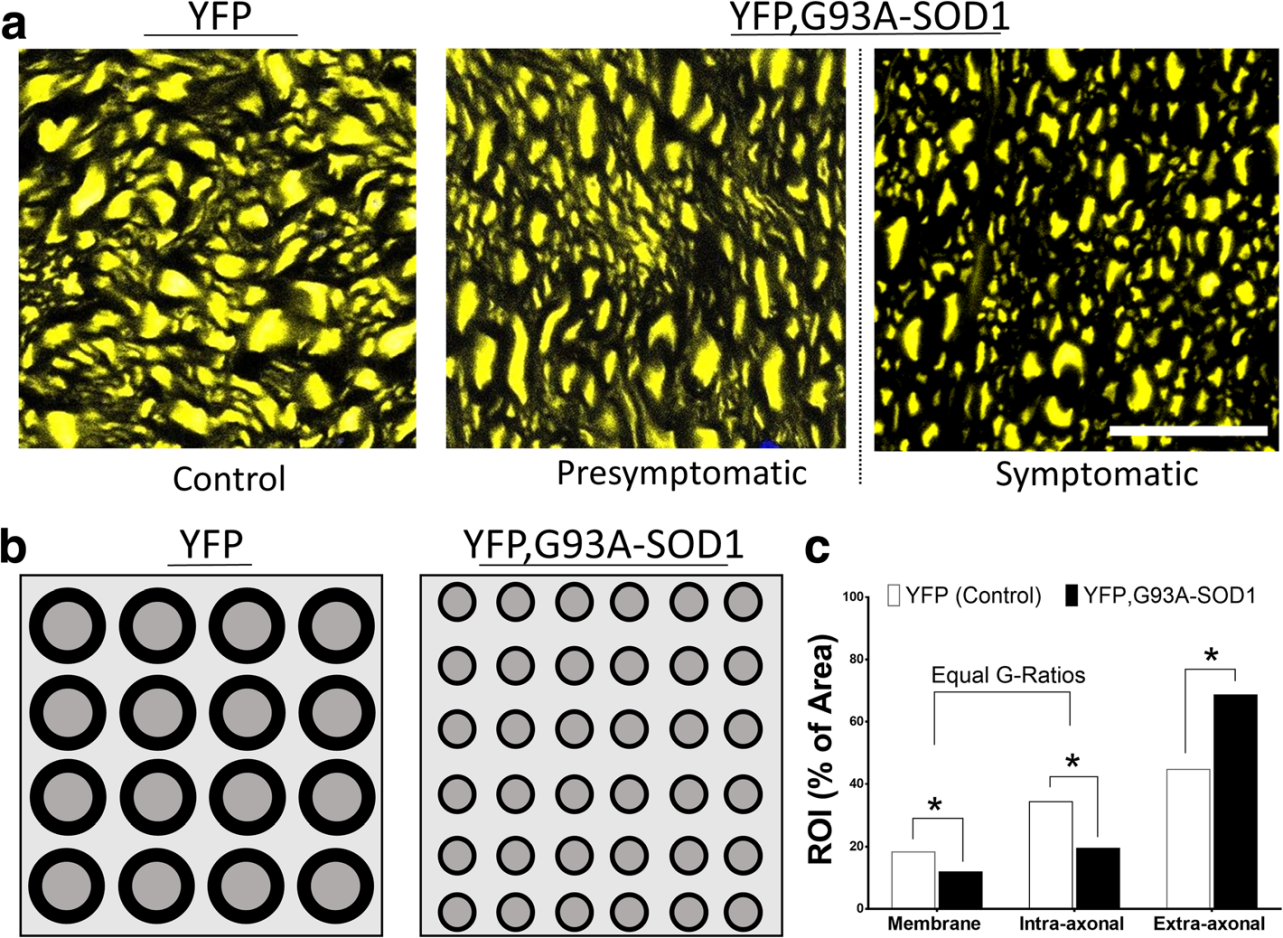
Diagram representing axonal structural changes detected by UHF-MRI and histology in ALS mice**. a** High magnification of axial section from spinal cord white matter (WM) in YFP control and YFP, G93A-SOD1 mice. **b** Diagram showing a reduction in axonal area and increased axonal density in the ALS mice. **c -** Graph displaying the relative changes in the membrane content (decrease in MBP described in Gatto et al. 2018 [31]), and relative decrease in axonal compartment and increase in the extra-axonal compartment. Note that concurrent changes in axonal compartment and membranes occurs without changes in G-ratios. Scale bar = 10 μm. Abbreviations: YFP, yellow fluorescent Protein; WM, white matter; GM: grey matter, MBP: myelin basic protein. AD: axial diffusion, RD: radial diffusion. G-ratio: ratio of the inner axonal diameter to the total outer diameter

Overall, this work proposes new insights to understand the biological nature of changes in MRI diffusion parameters during early stages of ALS (Fig. 6). Although previous MRI work has demonstrated a decrease in SC volume in ALS patients at symptomatic stages [77, 78], one of the limitations by our current MRI and histological techniques of axonal quantification is the exclusive assessment of ROIs without accounting for the volume reduction of the entire CNS structure (SC) interrogated. Moreover, recent findings using alternative diffusion techniques have demonstrated a similar increase in axonal densities during early stages of neurodegenerative diseases [79] pointing to an increase in the total number of axons per volume unit of WM tissues at early stage among different neurodegenerative diseases. Among the different mechanisms to explain the early structural changes, one is related to the specific susceptibility of subsets of neurons by the mutated G93A-SOD1 protein, producing an axonal dying-back mechanism [3, 41, 80]. Another theory is the relative impairment in the glial cell population [81], and both are considered the subject of future DTI in vivo studies.

Another limitation in the co-registration of UHF-MRI and histological techniques is the mismatch between the resolution of the MRI signal (100 μm) and the maximum optical resolution given by the laser confocal systems and fluorescent probe used in our biological preparations (1/2 the wavelength of the excitation light, where YFP = 0.534 microns). Hence, results from a single reconstructed tract from DTI data (Fig. 3a) represents an area of approx. 100-200 axons/voxel (Fig. 3b). Moreover, considering the overall WM axonal sizes (1-20 microns) and the mosaic expression of the YFP tag in this particular fluorescent animal (only 10-30% of the neuronal population) the current model clearly underrepresent the real number of axonal elements per voxel area (approx. 400-1000 axons/voxel).

Our studies have shown that UHF-MRI and DTI techniques are useful tools to detect early changes in the microstructural features of ALS (Table 2). Yet, our current techniques have some limitations due to the small range of b-values studied. In addition, the involvement of different cell populations as described in this work points towards the existence of an anomalous water diffusion process in a porous biological material marking the need for higher b-values and the use of more complex diffusion models [82–84] to evaluate the complex WM neuroanatomical changes occurring during the development of ALS [23]. Finally, more extensive neurobiological work should be done in order to validate each bioimaging marker during the early stages of this disease.

**Table 2:** Presymptomatic UHF-MRI Diffusion Bioimaging Markers at 17.6T and Histological Findings in ALS Mice Spinal Cord

MRI markers	White Matter Microstructure	Histology/Molecular Markers
FA ↓	Axonal Organization	YFP labeled axons
RD ↑	Myelin Content	MBP ↓*
AD ↓	Axonal Degeneration	YFP labeled axons
EW ↓	Axonal Connectivity	YFP labeled axons
ADC ↑	Transmembrane Water Diffusion	AQP4 **↑↑**

Presymptomatic white matter changes in UHF-MRI diffusion can be associated to biological and molecular markers of axonal ultrastructure & connectivity and other cellular compartments

Abbreviations: *ALS* Amyotrophic lateral Sclerosis, *WM* White matter, *ADC* Apparent diffusion coefficient, *RD* Radial Diffusivity, *AD* Axial diffusivity, *FA* Fractional Anisotropy, *EW* Edge Weight, *AQP4* Aquaporin 4, *MBP* Myelin Basic Protein, *YFP* Yellow fluorescent Protein. (* Presymptomatic changes in MBP seen in Gatto et al. 2018 [28])

## Conclusions

This work demonstrates that the combination of UHF-MRI and fluorescent mouse reporters is a useful approach to detect and characterize microstructural changes and axonal connectivity anomalies in ALS. Using a transgenic mouse fluorescent reporter, our studies were able to identify changes in axonal size, density and connectivity in the spinal cord of ALS mice before the disease was fully manifest. We found that anomalies in diffusion markers captured by DTI are possibly connected with alterations in cellular and molecular markers observed in our fluorescent ALS mice. We described early WM changes using connectomics as an additional method to evaluate early axonal connectivity anomalies in ALS. We believe this investigation is a step in the characterization of early bioimaging markers to detect and eventually monitor future treatments in patients with ALS.

## Abbreviations

AD: Axial diffusion
ALS: Amyotrophic lateral Sclerosis
AQP4: Aquaporin 4
DTI: Diffusion Tensor Imaging
FA: Fractional anisotropy
G93A-SOD1: transgenic mice with the overexpression of human mutant gene copper zinc superoxide dismutase identified in familiar forms of ALS patients
GM: Grey matter
G-ratio: ratio of the inner axonal diameter to the total outer diameter
RD: Radial diffusion
SC: Spinal Cord
UHF-MRI: Ultra**-**High Field MRI
WE: Weight edge connectomics
WM: White matter
